# Ethanol-Induced Hepatic Insulin Resistance is Ameliorated by Methyl Ferulic Acid Through the PI3K/AKT Signaling Pathway

**DOI:** 10.3389/fphar.2019.00949

**Published:** 2019-08-29

**Authors:** Qi Cheng, Yong Wen Li, Cheng Fang Yang, Yu Juan Zhong, Li Li

**Affiliations:** College of Pharmacy, Guilin Medical University, Guilin, China

**Keywords:** alcoholic liver disease, insulin resistance, methyl ferulic acid, PI3K/AKT pathway, glucose and lipid metabolism disorder

## Abstract

One of the key events during the development of alcoholic liver disease (ALD) is that alcohol inhibits the insulin signaling pathway in liver and leads to disorders of glucose and lipid metabolism. Methyl ferulic acid (MFA) is a biologically active monomer isolated from the root of *Securidaca inappendiculata* Hasskarl. It has been reported that MFA has a hepatoprotective effect against alcohol-induced liver injury *in vivo* and *in vitro*. However, the effect of MFA on ethanol-induced insulin resistance in ALD remains unclear. In this study, we investigated whether MFA could exert protective effects against hepatic insulin resistance in ethanol-induced L-02 cells and ALD rats. ALD was induced in vivo by feeding Lieber-DeCarli diet containing 5% (w/v) alcohol for 16 weeks to Sprague-Dawley rats. Insulin resistance was induced *in vitro* in human hepatocyte L-02 cells with 200 mM ethanol for 24 h followed by 10-7 nM insulin for 30 min. MFA exhibited the effects of inhibited insulin resistance, reduced enzymatic capacity for hepatic gluconeogenesis, and increased hepatic glycogen synthesis both *in vivo* and *in vitro*. In addition, the results of transcriptome sequencing of liver tissues in the ethanol- and MFA-treated groups indicated that “pyruvate metabolism,” “glycolysis/gluconeogenesis,” and “fatty acid metabolism” were significantly different between ethanol- and MFA-treated groups. Further studies suggested that MFA activated the hepatic phosphatidylinositol 3-kinase (PI3K)/AKT pathway *in vivo* and *in vitro*. Taken together, these findings suggested that MFA effectively ameliorated hepatic insulin resistance in ALD at least partially by acting on the PI3K/AKT pathway.

## Introduction

Recent epidemiological studies have shown that ethanol abuse has become one of the major determinants of chronic noncommunicable diseases such as alcoholic liver disease (ALD), cardiovascular disease, and type 2 diabetes mellitus (T2DM) (Parry et al., 2011). At present, the relationship between alcohol and diabetes has received widespread attention. It has been reported that alcohol consumption has a U-shaped or J-type correlation with the risk of T2DM, that is, moderate drinkers have a lower risk of developing T2DM, whereas excessive drinkers have a high risk (Baliunas et al., 2009; Knott et al., 2015; Li et al., 2016). Insulin resistance is the main characteristic and influential factor of T2DM, which is characterized by a decrease in the ability of insulin to take up and eliminate glucose from the surrounding tissues, resulting in a disorder of glucose metabolism (Szkudelski and Szkudelska, 2015). There is mounting evidence from recent epidemiological studies that excessive alcohol consumption induces insulin resistance (Couzigou et al., 2008; Schrieks et al., 2015).

The liver, as the body’s largest endocrine organ and the main target organ of insulin and alcohol, plays an important role in regulating blood glucose homeostasis. Hepatic glucose metabolism is very complex and is regulated by multiple pathways, including the phosphatidylinositol 3-kinase (PI3K)/protein kinase B (AKT) signaling pathway (Sharma et al., 2015). Under normal circumstances, insulin binds to insulin receptor (IR) on the surface of the hepatocyte membrane and activates the autophosphorylation of protein tyrosine kinase on the β-subunit. Activated IR activates PI3K *via* the phosphorylation of IR substrate (IRS) protein (Zhang et al., 2014). PI3K is a phosphatidylinositol kinase that contains a catalytic subunit (p110) and a regulatory subunit (p85). Activated PI3K catalyzes the conversion of phosphatidylinositol 4,5-diphosphate (PIP2) to phosphatidylinositol 3,4,5-triphosphate (PIP3), which is the main substrate of phosphatase and tensin homologue deleted on chromosome 10 (PTEN) (He et al., 2010). Previous research has shown that PTEN converts PIP3 back to PIP2 through its phosphatase activity to negatively regulate insulin signalling (Ruan et al., 2009). PIP3 is a key second messenger that catalyzes the activation of AKT autophosphorylation. In the liver, activated AKT has four main roles (Carr and Correnti, 2015): 1) stimulation of glycogen production by inhibiting glycogen synthase (GS) kinase (GSK) to increase GS activity (Beurel et al., 2015); 2) suppression of gluconeogenesis in part by inactivating forkhead box O1 (FoxO1) to decrease the expression of key gluconeogenic genes, such as phosphoenolpyruvate carboxykinase (PEPCK), glucose-6-phosphatase (G-6-Pase), and fructose 1,6-bi-phosphatase (FBPase) (Gross et al., 2009; Naowaboot et al., 2016; Ghanem et al., 2017); 3) stimulation of endogenous fatty acid synthesis by regulating sterol regulatory element-binding protein 1 (SREBP1) (Shao and Espenshade, 2012); and 4) promoting glucose transporter 2 (GLUT2) to transport glucose from the periphery into the cells for aerobic metabolism or anaerobic degradation (Xuguang et al., 2019). Previous studies have shown that ethanol can in part induce hepatic insulin resistance through the inhibition the PI3K/AKT pathway by decreasing IR density, inhibiting the binding affinity between insulin and its receptor, and decreasing receptor phosphorylation (Pang et al., 2009; Gunji et al., 2011).

The drugs currently used to improve insulin resistance mainly include biguanide and thiazolidinedione. Metformin, which is representative of biguanide drugs, is limited by the development of gastrointestinal side effects. In addition, rosiglitazone has been withdrawn due to an increased cardiovascular risk (Nissen and Wolski, 2007). Furthermore, pioglitazone is also limited due to the potential to increase the risk of bladder cancer (Lewis et al., 2011). Therefore, the clinically available drugs for the treatment of insulin resistance have become more limited. In recent years, numerous Chinese herbal medicines and their active ingredients have been found to ameliorate insulin resistance, such as ginseng, resveratrol, and *Pueraria lobata* root (Lee et al., 2012; Seong et al., 2016; Chen et al., 2018), and they have fewer side effects than synthetic drugs. Hence, Chinese herbal medicine for the prevention and treatment of insulin resistance has become a research hotspot domestically and overseas. Methyl ferulic acid (MFA) is a white needle compound that possesses a variety of pharmacological properties, including antioxidant stress, anti-apoptosis, and anti-inflammatory effects (Li et al., 2017; Cheng et al., 2018; Li et al., 2018; Yang et al., 2018; Cheng et al., 2019). However, to our knowledge, there are still no reports concerning the effect of MFA on insulin resistance at home and abroad. In the present research, we established ethanol-induced rat and cell models of insulin resistance to investigate whether MFA had a therapeutic effect on insulin resistance and explored its effects on hepatic gluconeogenesis and glycogen synthesis.

## Experimental Procedures

### Chemicals and Reagents

MFA, described as 3,4-dimethoxy cinnamic acid (>98% purity), was purchased from Sigma Chemical Co. (St. Louis, MO, USA). MFA was dissolved in dimethyl sulfoxide (DMSO; Xilong Chemical Co., Ltd., Guangdong, China) for cell experiments *in vitro* and in starch paste for intragastric administration to rats *in vivo*. Absolute ethanol was purchased from Zhiyuan Chemical Reagent Co., Ltd. (Tianjin, China). The immunoprecipitation (IP) kit was obtained from Sangon Biotech Co., Ltd. (Shanghai, China). Recombinant human insulin injection was obtained from DONGBRD Pharmaceutical Co., Ltd. (Tonghua, China). Primary antibodies against IR, p-IR (Y1185), p-IRS1 (Y896), p-IRS2 (S731), PI3K-p85, PTEN, p-AKT2 (S474), GSK3β, p-GSK3β (Y216), FoxO1, p-FoxO1 (S256), SREBP1, p-GS (S641), and GS were purchased from Abcam Technology (Cambridge, UK). Primary antibodies against AKT1, p-AKT1 (Ser473), PEPCK, and AKT2 were obtained from Sangon Biotech. The primary antibody against PI3K-p110 was purchased from Cell Signaling Technology (Beverly, MA, USA). The primary antibody against G-6-Pase was obtained from Santa Cruz Biotechnology (Santa Cruz, CA, USA). Primary antibody against FBPase was purchased from Roche (Mannheim, Germany). Primary antibody against GLUT2 and β-actin were obtained from ZSGB Biotech Co., Ltd. (Beijing, China). All other reagents were purchased from Sigma unless indicated otherwise.

### Animals and Protocols

Sixty adult male Sprague-Dawley (SD) rats (180–220 g) were purchased from the Experimental Animal Centre of Guilin Medical College (Guilin, China) and maintained under standard living conditions (room temperature of 22 ± 2°C, 50–60% relative humidity, and 12 h dark/light cycle). All experimental procedures were conducted in accordance with the Chinese legislation and the U.S. National Institutes of Health guidelines for the care and use of experimental animals, and the animal experiments were approved by the Institutional Ethical Committee of Guilin Medical University.

After acclimatization for 1 week, all rats were randomly separated into five groups (12 rats per group): 1) control, 2) ethanol, 3) ethanol + MFA (20 mg/kg/day), 4) ethanol + MFA (10 mg/kg/day), and 5) ethanol + MFA (5 mg/kg/day). Ethanol-induced rats were fed Lieber-DeCarli liquid diet for 16 weeks, whereas control normal rats were given isothermal liquid diet containing dextrin maltose. As mentioned earlier, ethanol was gradually introduced for 1 week before providing rats with a final concentration (5% v/v) that represented the maintenance dose of 36% of the total calories (Lee et al., 2011). All liquid diets were freshly prepared before use. Rats in the MFA groups received intragastric administration daily for the last 4 weeks, whereas rats in the control and ethanol groups were given an equal amount of vehicle. Food and water were available *ad libitum*, and body weights were recorded weekly throughout the experiment. After 16 weeks, all rats were fasted overnight and sacrificed by spinal dislocation. Blood samples were collected in heparin-containing tubes by centrifugation (3,000 × g, 4°C) for 20 min and stored at −80°C until analysis. Liver and spleen tissues were rapidly excised, rinsed, with physiological saline solution, dried with filter paper, and weighed. A portion of liver was fixed, and the rest was stored at −80°C until analysis.

### Detection of the Visceral Index

The liver and spleen indices were calculated according to the following method: Liver index (%) = (Liver weight/Body weight) × 100% and Spleen index (%) = (Spleen weight/Body weight) × 100%.

### Serum Transaminase Activity Measurements

Plasma alanine aminotransferase (ALT) and aspartate aminotransferase (AST) levels were measured as indicators of liver function. The levels of ALT and AST were measured using commercially available diagnostic kits (Guilin Elite Medical Electronics Co., Ltd., Guilin, China) by the Erba XL-600 automatic biochemistry analyzer (ERBA Diagnostics Mannheim GmbH, Mannheim, Germany).

### Liver Histology

Liver pathological examination was performed using hematoxylin and eosin (H&E) staining as described previously (Bai et al., 2014). Liver tissues were fixed in 10% neutral-buffered formalin solution for 24 h. After processing by conventional histological procedures, sections with a thickness of 5 µm were cut, dewaxed in xylene, and rehydrated in an alcohol gradient. After H&E staining, the slides were observed under a light microscope (Olympus BX41, Olympus, Tokyo, Japan) for general morphological evaluation and photographed at 100× magnification.

### Serum Glucose and Insulin Measurements

To measure serum glucose and insulin, rats were fasted overnight, and blood samples were collected and tested. The fasting serum glucose levels were detected using commercially available diagnostic kits (Guilin Elite Medical Electronics) by an automatic biochemical analyzer. Fasting serum insulin levels were determined using an ultra-sensitive mouse insulin immunoassay kit (Huamei Biotech Co., Ltd., Wuhan China) according to the manufacturer’s instructions. The homeostasis model assessment of insulin resistance (HOMA-IR) was calculated according to the following formula (Zhao et al., 2010): fasting glucose (mmol/L) × fasting insulin (µIU/ml)/22.5.

### Hepatic Glycogen Content and Triglyceride (TG) Level Determination

The content of hepatic glycogen was determined using a liver/muscle glycogen assay kit (Nanjing Jiancheng Bioengineering Institute, Nanjing, China) based on the anthrone reagent method. The hepatic TG levels in approximately 100 mg liver tissues homogenized to extract lipids were determined using a TG quantification kit (Guilin Elite Medical Electronics) by an automatic biochemical analyzer.

### Transcriptome Sequencing of Liver Tissues in Ethanol-Induced Rats

After the model of ALD was successfully established in rats, three rats were randomly selected from the ethanol group and the ethanol + MFA 10 mg/kg group. Then, the liver tissues were collected for transcriptome sequencing. Sequencing was performed at MH BioTech Co., Ltd. (Shanghai, China) on an Illumina HiSeqTM 2000. Gene Ontology (GO) analysis and pathway enrichment analysis were performed using the sequencing data.

### Cell Culture and Treatment

Human hepatocyte L-02 cells (human normal liver cells; no. GDC079) were purchased from the China Centre for Type Culture Collection of Wuhan University (Wuhan, China). L-02 cells were grown in Dulbecco’s modified Eagle’s medium (DMEM; Thermo Fisher, MA, USA) supplemented with 10% (v/v) fetal bovine serum (Sangon Biotech) and 1% antibiotic solution (100 units/ml penicillin and 100 μg/ml streptomycin). L-02 cells were maintained in a humidified incubator with a 5% CO2/95% air atmosphere at 37°C and passaged daily by trypsin-EDTA digestion (Solarbio Co., Beijing, China). The insulin-resistant cell model was established as described previously with some modifications (Chen et al., 2015). After attachment to the plates, L-02 cells were washed three times with phosphate-buffered saline (PBS) and starved in serum-free medium for 12 h. The cells were then cultured in medium as the control group, in medium plus 200 mM ethanol as the model group, and in medium plus 200 mM ethanol in the presence of 100, 50, or 25 μM MFA as the MFA treatment groups for 24 h. Before harvest, L-02 cells were subsequently treated with insulin (100 nM) for 30 min.

### 3-(4,5-Dimethylthiazol-2-yl)-2,5-Diphenyltetrazolium Bromide (MTT) Assay

The cell viability of MFA on L-02 cells was determined using the MTT (Sigma) assay. Briefly, L-02 cells were plated in 96-well plates (1 × 104 per well). After attachment to the plates, L-02 cells were treated with 12.5, 25, 50, 100, 200, 400, and 800 μM MFA for 24 h. After treatment, 20 μL MTT (final concentration 0.5 mg/ml) was added to each well, and the plates were incubated for 4 h at 37°C. After incubation, the medium was removed, and 150 μL DMSO was added to each well to dissolve the formazan crystals. The optical density (OD) was measured at 490 nm using a microplate reader (Bio-Rad, Inc., Hercules, CA, USA). The percentage of cell viability was calculated as follows:

Cell viability (%) = [(A_490, Sample_ − A_490, Blank_)/(A_490, Control_ − A_490, Blank_)] × 100%.

### Glucose Consumption Assay

Glucose consumption was measured using a commercial glucose assay kit according to the manufacturer’s instructions. Briefly, L-02 cells (7 × 10^4^ per well) were cultured in a 24-well plate, and after starvation in serum-free DMEM for 12 h, the medium was replaced with DMEM plus 200 mM ethanol and/or MFA (100/50/25 μM) for 24 h followed by incubation in medium plus 100 nM insulin for 30 min. The glucose concentration in the supernatant was measured by an Erba XL-600 automatic biochemical analyzer using the glucose assay kit (Guilin Elite Medical Electronics) and normalized to the total cellular protein. Glucose consumption was calculated by the glucose concentration of blank wells minus the glucose concentration in the plated wells.

### Intracellular Glycogen Content and TG Level Determination

L-02 cells were cultured in 10-cm-diameter Petri dishes (3 × 10^6^ per plate). After serum starvation treatment for 12 h, the medium was replaced with DMEM plus 200 mM ethanol and/or MFA (100/50/25 μM) for 24 h followed by incubation in medium plus 100 nM insulin for 30 min. The medium was then discarded, and the cells were washed twice with PBS. The glycogen content of L-02 cells was determined using commercial kits (Nanjing Jiancheng Bioengineering Institute) based on the anthrone reagent method. The TG levels of L-02 cells were determined using a TG quantification kit (Guilin Youlitt Medical Electronics Co., Ltd., Guilin, China) by an automatic biochemical analyzer.

### Western Blot Analysis

The total proteins from L-02 cells and rat liver tissues (100 mg) were homogenized in ice-cold radioimmunoprecipitation assay buffer (Beyotime Bio Co., Nanjing, China) containing protease inhibitor cocktail (×100) and phosphatase inhibitors (Sangon Biotech). The supernatants were collected by centrifugation at 13,000 rpm for 20 min at 4°C. Protein concentrations were assessed using a modified-form BCA protein concentration assay kit (Beyotime Bio). Protein samples were mixed with sample buffer and denatured by boiling for 5 min at 100°C. Equal amounts (50 µg) of sample protein were separated by electrophoresis in a sodium dodecyl sulfate-polyacrylamide gel electrophoresis and transferred onto nitrocellulose membranes (Pall Co., NY, USA). Next, the membranes were blocked in 5% non-fat powdered milk or bovine serum albumin in Tris-buffered saline Tween 20 buffer for 1 h at room temperature and incubated with the specific primary antibodies overnight at 4°C. After washing four times, the membranes were incubated with the secondary antibody (Sangon Biotech) for 1 h at room temperature to reveal the bands with enhanced chemiluminescence reagent (Tsea Biotech Co., Shanghai, China). The Molecular Imager Chemi Doc XRS (Bio-Rad) and JS-780 automatic gel imaging analysis system were used to reveal the bands and to perform quantitative analysis of the blots. Parallel blotting of β-actin was used as an internal control.

### IP Assay

To study the relationship among PI3K-p85, PI3K-p110, and PTEN, an IP assay was performed according to the manufacturer’s approach. Liver tissues or L-02 cell lysates were homogenized in ice-cold lysis buffer containing protease and phosphatase inhibitors as mentioned earlier. After centrifugation at 12,000 rpm for 5 min, the supernatant was collected as a cell/tissue lysate. Anti-PI3K-p85 antibody (1 µg) was added to the total cell/tissue lysates in a 2 ml centrifuge tube and incubated for 1 h at 4°C. The total protein lysate was added to protein A Sepharose beads (14 µl), immunoprecipitated for 2 h at 4°C, and spun at 12,000 × g for 30 s at 4°C. After washing three times with IP buffer, 50 µl sample buffer was added and boiled for Western blotting with the indicated antibodies.

### RNA Isolation and Reverse Transcription-Polymerase Chain Reaction (RT-PCR)

Total RNA from L-02 cells and liver tissue samples was isolated and extracted using a tissue/cell total RNA isolation kit (Tiangen Biotech Co., Ltd., Beijing, China) according to the manufacturer’s protocol. Total RNA was reversibly transcribed to cDNA using a cDNA synthesis kit (TIANScript cDNA; Tiangen Biotech) according to the manufacturer’s approach. The MJ PTC-200 PCR system (Bio-Rad) and RT-PCR kit (2× Taq PCR Master Mix, Aidlab Biotech Co., Ltd., Beijing, China) were used for RT-PCR. Specific primers for genes (**Table 1**) were provided by Sangon Biotech. The PCR protocol was as follows: pre-denaturation for 4 min at 94°C for 1 cycle; denaturation for 30 s at 94°C, annealing for 30 s at 50°C to 60°C, and extension for 1 min at 70°C for 32 cycles; and an additional extension for 5 min at 72°C. The PCR products were identified using 2% agarose gel electrophoresis. The OD of the target gene band of each sample was calculated using a Gel Doc XR^+^ automatic gel imaging analysis system (Bio-Rad) and adjusted by β-actin correction to obtain the relative expression of the target gene.

**Table 1:** Primer sequences used for the determination of PEPCK, G-6-Pase, GS, and β-actin gene expression.

Species	Gene	Forward primer (5′ → 3′)	Reverse primer (5′ → 3′)
Human	β-actin	TTTTGGCTATACCCTACTGGCA	CTGCACAGTCGTCAGCATATC
	PEPCK	TTGAGAAAGCGTTCAATGCCA	CACGTAGGGTGAATCCGTCAG
	G-6-Pase	GTGTCCGTGATCGCAGACC	GACGAGGTTGAGCCAGTCTC
	GS	TTTATGGGCATCTGGACTTCAAC	CGCTGCCGTTCACTCTGAG
	SREBP1	CGGAACCATCTTGGCAACAGT	CGCTTCTCAATGGCGTTGT
Rat	β-actin	TCCGGCACTACCGAGTTATC	CCTTGATCCGGTGTAGCAGAT
	PEPCK	TGACAGACTCGCCCTATGTG	CCCAGTTGTTGACCAAAGGC
	G-6-Pase	CGACTCGCTATCTCCAAGTGA	GTTGAACCAGTCTCCGACCA
	GS	TCCTGGCCCAGAACGAAGA	TGAGTGGTGAAGATGGTTGCC
	SREBP1	GATGTGCGAACTGGACACAG	CATAGGGGGCGTCAAACAG

### Statistical Analysis

All data are presented as mean ± SD. SPSS software (version 18.0) was used for statistical analysis. Significant differences between groups were evaluated using one-way analysis of variance. The Student’s-Newman-Keuls’ test was used for comparison between groups. Differences between groups were considered statistically significant at *P* < 0.05 and are indicated by different letters.

## Results

### MFA Improved Liver Injury in Alcohol-Induced Rats

**Figure 1:**
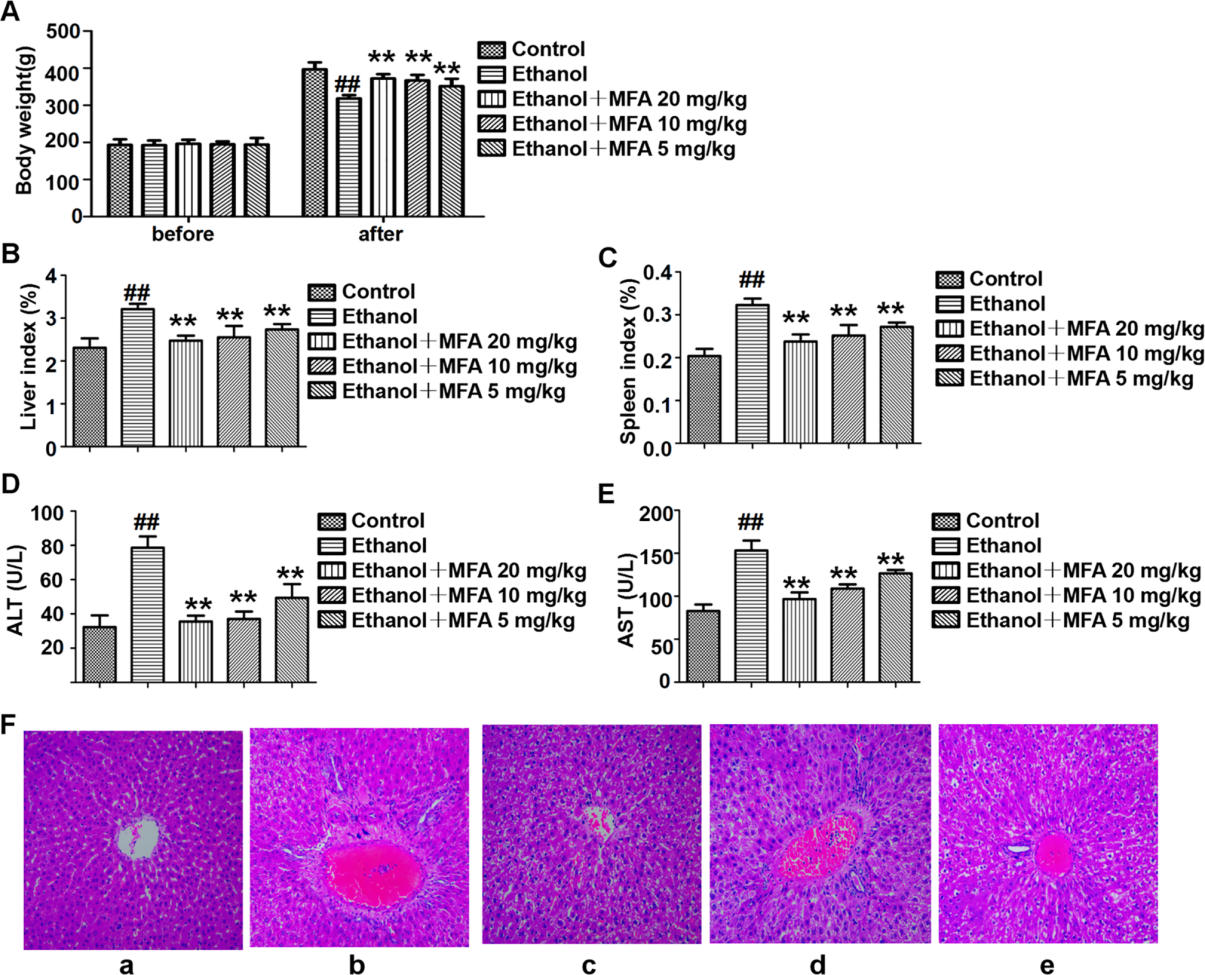
Effects of MFA on hepatic impairment in ALD rats. **(A)** Body weights of rats before the experiment and before being killed. **(B)** Liver index. **(C)** Spleen index. **(D)** Serum ALT levels. **(E)** Serum AST levels. Data are mean ± SD of at least three separate experiments (n = 8). ^##^*p* < 0.01, compared to the control group; **p < 0.01, compared to the ethanol group. **(F)** Histological analysis of liver tissue sections in each group. H&E staining was performed (magnification, ×100). **(a)** Control group. **(b)** Ethanol group. **(c)** Ezthanol + MFA 20 mg/kg group. **(d)** Ethanol + MFA 10 mg/kg group. **(e)** Ethanol + MFA 5 mg/kg group.

As shown in **Figure 1A**, there were no obvious differences in rat initial body weight among the five groups. Rats fed Lieber-DeCarli liquid diet for 16 weeks had markedly lower body weight than those in the control group (*p* < 0.01). Compared to the ethanol group, the body weight of these ethanol-fed rats was significantly increased after 4 weeks of MFA treatment (*p* < 0.01). Ethanol-induced rats had notably elevated liver and spleen indices, which were obviously down-regulated under MFA treatment in a dose-dependent manner (*p* < 0.01; **Figures 1B, C**).

Next, serum biomarkers of hepatic function were measured to examine whether MFA attenuated ethanol-induced liver injury. The results showed that ethanol-induced increase in serum ALT and AST levels was significantly attenuated by MFA treatment (*p* < 0.01; **Figures 1D, E**). In addition, to study histopathological changes in ethanol-induced rats, H&E staining was performed with the representative micrograph shown in **Figure 1F**. Rats in the control group showed a normal liver morphology. Rats in the ethanol group showed significant hepatic pathological lesions. The hepatocyte arrangement was irregular, the structure of normal hepatic lobules was severely destroyed, and the arrangement of hepatic cords was disordered. Numerous fat droplets in hepatocytes, extensive collagen deposition, apoptosis, and widespread hepatocellular necrosis were also observed. The hepatic pathological changes induced by ethanol were partially reversed by MFA treatment. These results indicated that MFA had a therapeutic effect on liver injury in ethanol-induced rats.

### Comparison of Hepatic Transcriptome Profiling in Rats Between the Ethanol Group and the MFA 10 mg/kg Group

**Figure 2:**
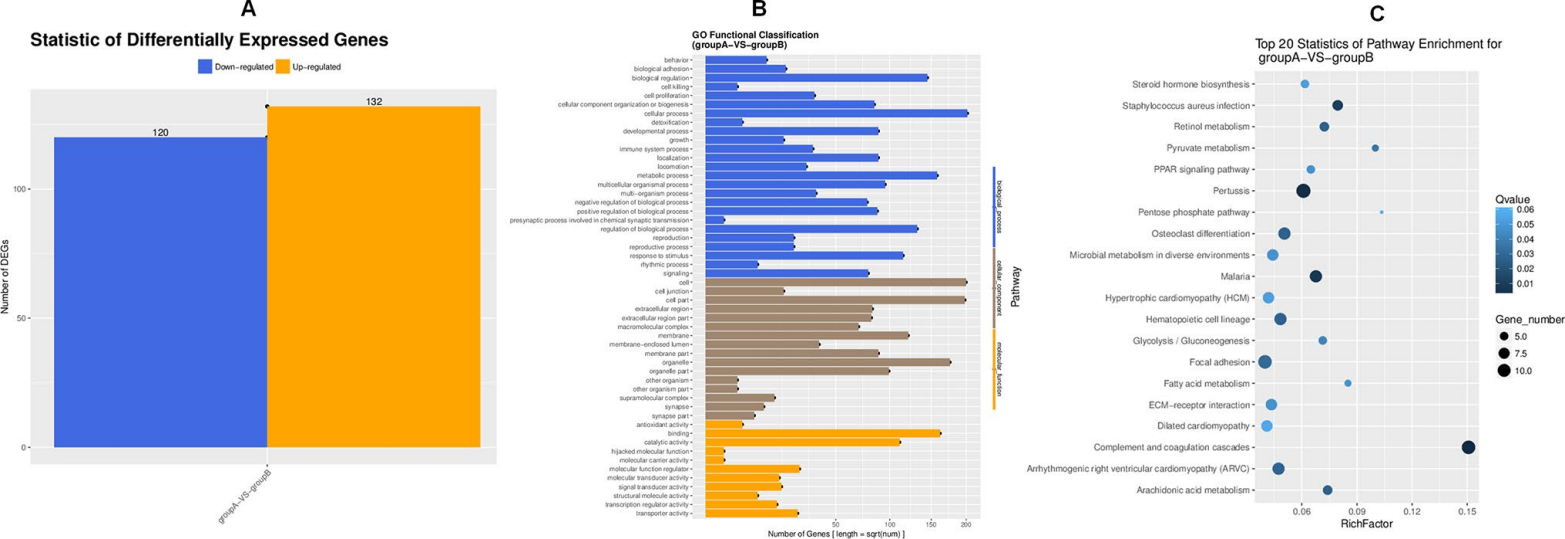
Transcriptome sequencing of liver tissues from rats in the ALD group and the MFA treatment group. **(A)** DEGs for group A versus group B. Vertical axis, number of DEGs; horizontal axis, group A versus group B. Group A is the ethanol group, and group B is the mid-dose MFA group. **(B)** GO functional classification of DEGs for group A versus group B. Annotation statistics of DEGs are shown in the secondary node of three categories of GO. Vertical axis, secondary nodes of three categories in GO; horizontal axis, number of genes. Group A is the ethanol group, and group B is the mid-dose MFA group. **(C)** Scatter plot of KEGG pathway enrichment statistics. The top 20 statistics for pathway enrichment for group A versus group B are shown. Vertical axis, pathway; horizontal axis, enriched factor. Group A is the ethanol group, and group B is the mid-dose MFA group.

To search for the key molecules influencing or forecasting the prognosis of MFA, we compared the global transcriptome profiles between the ethanol group and medium MFA dose group by performing mRNA sequencing and bioinformatic analysis. As shown in **Figure 2A**, there were 252 differentially expressed genes (DEGs) between the ethanol group and the MFA group, among which 120 genes were down-regulated and 132 genes were up-regulated. A total of 252 DEGs were performed using GO annotation according to the DAVID dataset. They were distributed into three main ontologies, i.e., biological process, molecular function, and cellular component, and most DEGs were enriched in the biological process category. Comparing the medium MFA dose group to the ethanol group (**Figure 2B**), the highest proportions of DEGs were distributed in the cellular process subcategory in the biological process category. The down-regulated/up-regulated DEGs were pooled, and pathway analysis was conducted according to the KEGG dataset. The results are shown in **Figure 2C**. The top 20 statistics of pathway enrichment between the ethanol group and the MFA group included “Pyruvate metabolism,” “Glycolysis/Gluconeogenesis,” and “Fatty acid metabolism,” suggesting that MFA can improve ethanol-induced ALD through glycolysis/gluconeogenesis and fatty acid metabolism.

### MFA Alleviated Insulin Resistance in Ethanol-Induced Rats

**Figure 3:**
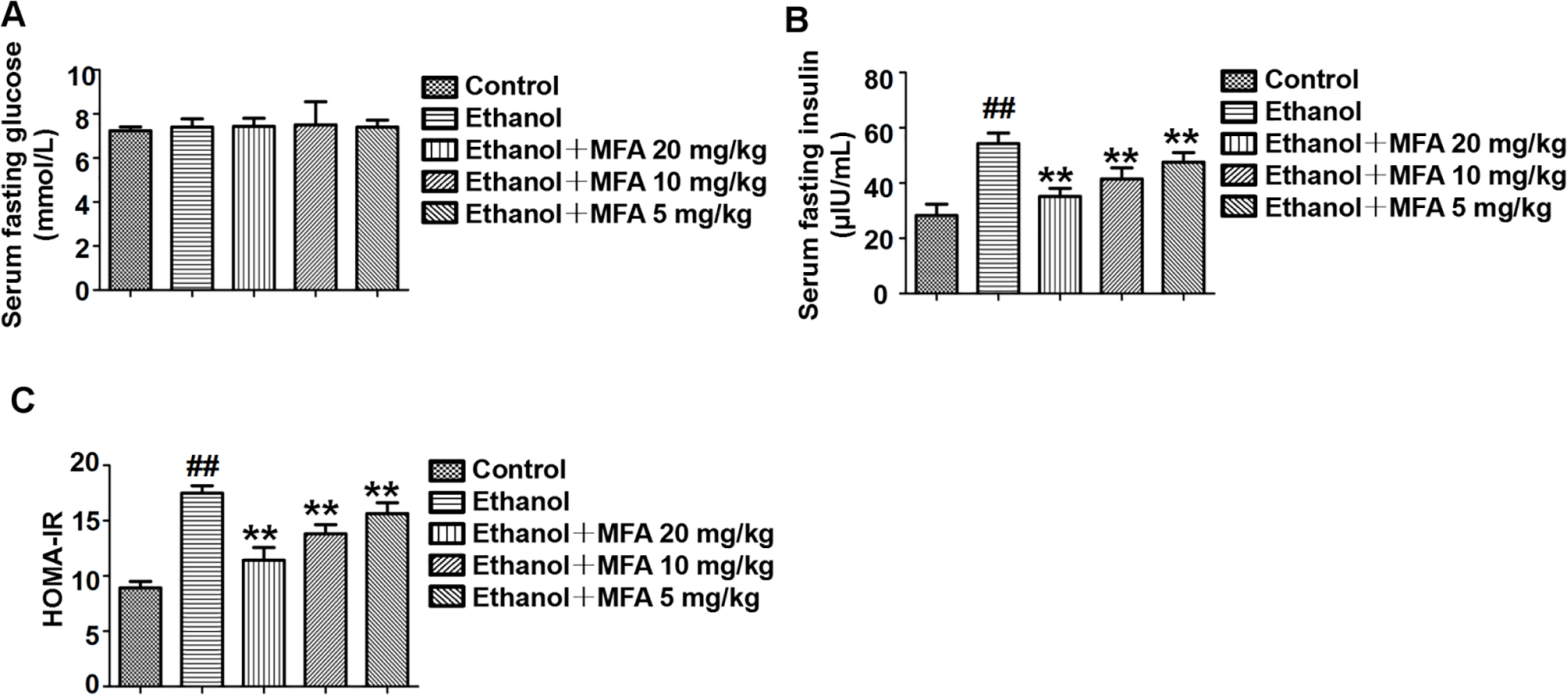
Effects of MFA on insulin resistance in ethanol-induced rats. **(A)** Fasting serum glucose levels. **(B)** Fasting serum insulin concentrations. **(C)** HOMA-IR index. Data are mean ± SD of at least three separate experiments (n = 8). ^##^ *p* < 0.01, compared to the control group; **p < 0.01, compared to the ethanol group.

In the present study, we used the HOMA-IR to assess insulin resistance in ethanol-induced rats. As shown in **Figure 3A**, there were no significant differences in fasting serum glucose levels in rats among the five groups. Furthermore, the fasting serum insulin levels in ethanol-induced rats were notably higher than these in normal rats (*p* < 0.01). Interestingly, the fasting serum insulin levels were significantly decreased after MFA treatment (*p* < 0.01; **Figure 3B**). Correspondingly, HOMA-IR was significantly increased in the ethanol group compared to the control group, which was notably decreased by MFA treatment in a dose-dependent manner (*p* < 0.01; **Figure 3C**). Taken together, these results indicated that MFA had a protective effect against ethanol-induced insulin resistance in rats.

### MFA Ameliorated the Insulin Signaling Pathway in Ethanol-Induced Rats

**Figure 4:**
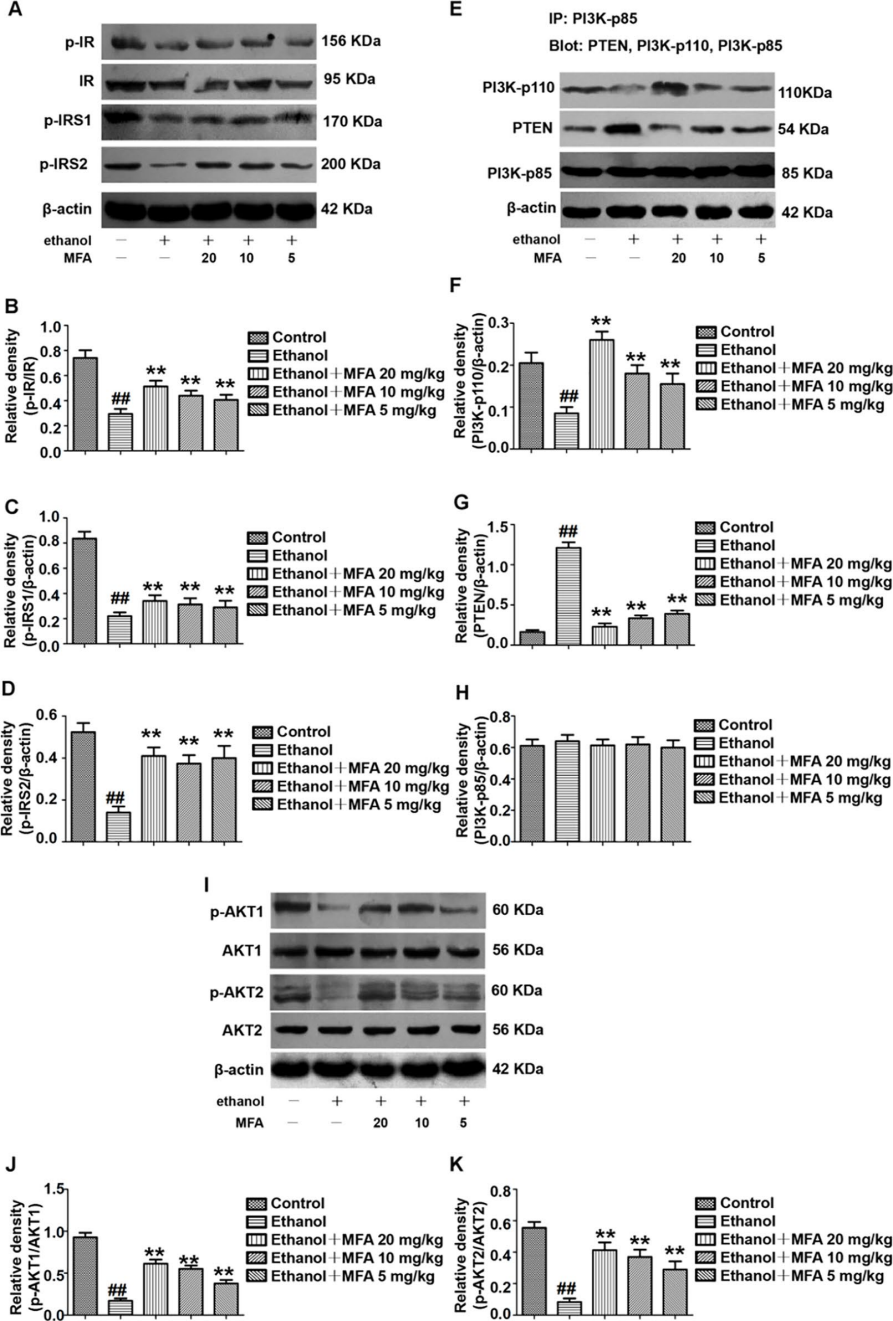
Effects of MFA on hepatic impairment of the insulin signaling pathway in ethanol-induced rats. **(A)** Representative immunoblots of IR, IRS1, and IRS2 in rat liver tissues. **(B)** Levels of IR phosphorylation. **(C)** Levels of IRS1 phosphorylation. **(D)** Levels of IRS2 phosphorylation. **(E)** Representative immunoblots of PI3K and PTEN in rat liver tissues. **(F)** Levels of PI3K-p110. **(G)** Levels of PTEN. **(H)** Levels of PI3K-p85. **(I)** Representative immunoblots of AKT1 and AKT2 in rat liver tissues. **(J)** Levels of AKT1 phosphorylation. **(K)** Levels of AKT2 phosphorylation. Data are mean ± SD of at least three separate experiments (n = 3). ^##^*p* < 0.01, compared to the control group; **p < 0.01, compared to the ethanol group.

To determine whether MFA could alleviate the damage to the hepatic insulin signaling pathway in ethanol-induced rats, we examined the protein expression levels of related genes in the hepatic insulin signaling pathway by Western blotting. The protein expression levels of p-IR, p-IRS-1, and p-IRS-2 were significantly decreased in ethanol-induced rats, which were all restored by MFA treatment (*p* < 0.01; **Figures 4A–D**).

We also assessed the protein expression levels of major genes in the PI3K/AKT signaling pathway. We examined the effects of PI3K-p85, PI3K-p110, and PTEN on the PI3K-AKT signaling pathway in the liver using the IP assay. The protein expression levels of PI3K-p85 in rats were unchanged when induced by ethanol as well as MFA. In the livers of ethanol-induced rats, ethanol significantly decreased PI3K-p110 protein expression levels in PI3K-p85 when PI3K-p85 protein was immunoprecipitated and then blotted using PI3K-p110 antibody (*p* < 0.01). In contrast, when PI3K-p85 protein was immunoprecipitated and then blotted using PTEN antibody, the PTEN protein expression levels in PI3K-p85 were higher in the livers of rats induced by ethanol (*p* < 0.01). Interestingly, these effects of ethanol were reversed after MFA administration (*p* < 0.01), indicating that MFA promoted the binding of PI3K-p110 to PI3K-p85 and inhibited the binding of PTEN to PI3K-p85 (**Figures 4E–H**).

Moreover, the protein expression levels of p-AKT1 and p-AKT2 in rats were significantly reduced in the ethanol group compared to the control group (*p* < 0.01). However, MFA treatment notably increased the expression levels of p-AKT1 and p-AKT2 (*p* < 0.01; **Figures 4I–K**). Collectively, these results indicated that MFA had a positive effect on the hepatic impairment of the insulin signaling pathway in ethanol-induced rats.

### MFA Up-Regulated Hepatic Glycogen Synthesis and GLUT in Ethanol-Induced Rats

**Figure 5:**
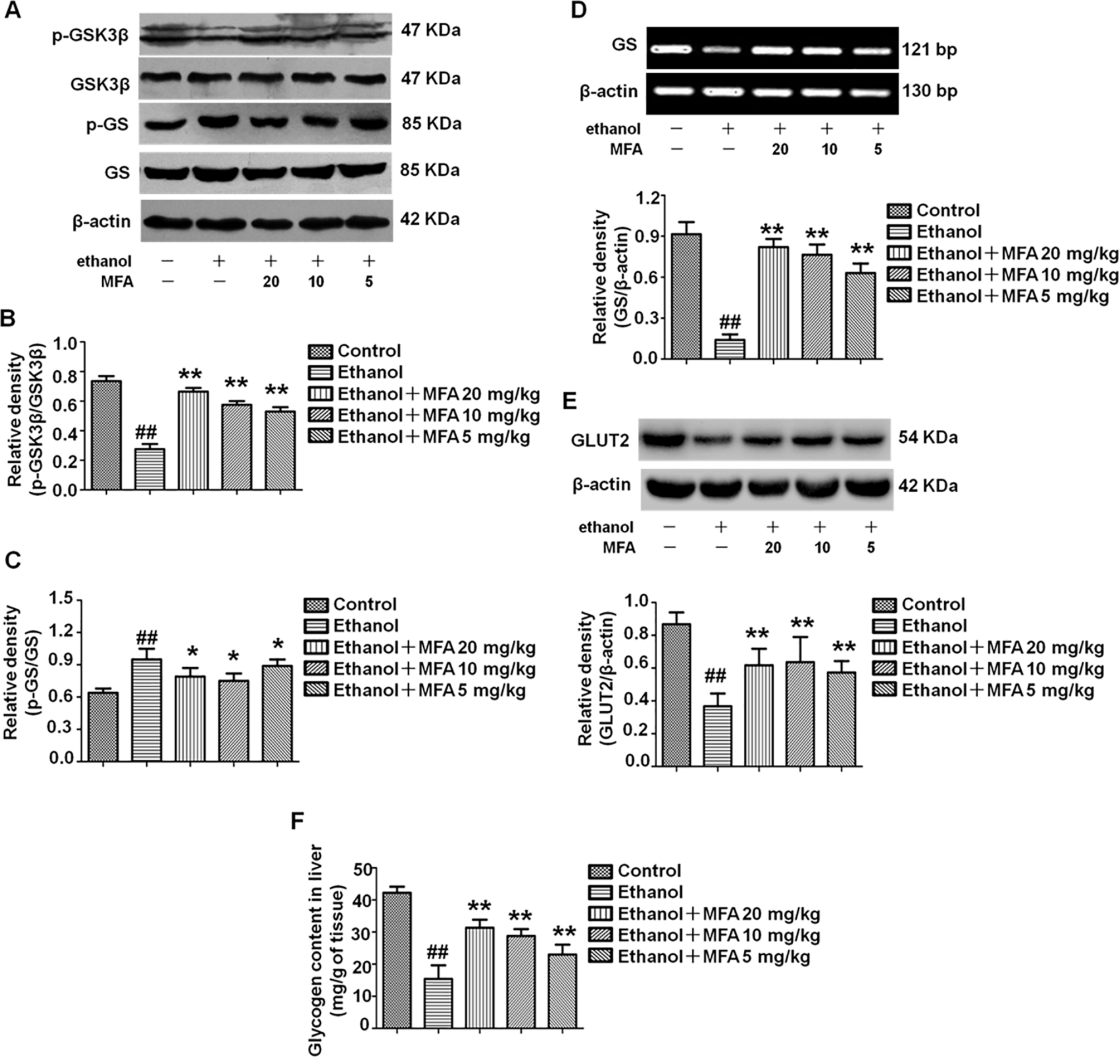
Effects of MFA on hepatic glycogen synthesis and GLUT2 in ethanol-induced rats. **(A)** Representative immunoblots of GSK3β and GS in rat liver tissues. **(B)** Levels of GSK3β phosphorylation. **(C)** Levels of GS phosphorylation. **(D)** mRNA expression of GS in rat liver tissues. **(E)** Protein expression of GLUT2. **(F)** Glycogen content in liver tissues. Data are mean ± SD of at least three separate experiments (n = 3). ^##^*p* < 0.01, compared to the control group; *p < 0.05, **p < 0.01, compared to the ethanol group.

In the present study, ethanol exposure significantly inhibited the protein expression levels of p-GSK3β and enhanced the protein expression levels of p-GS in rats, which were both reversed by MFA treatment (*p* < 0.01 or 0.05; **Figures 5A–C**). However, the expression levels of GS and GLUT2 were decreased in ethanol-induced rats, which could be increased by MFA treatment (*p* < 0.01; **Figures 5D, E**). In addition, we also assessed the hepatic glycogen content in rats. As shown in **Figure 5F**, ethanol-induced rats displayed a reduced content of hepatic glycogen, whereas the application of MFA up-regulated this reduction (*p* < 0.01). Taken together, these results suggested that MFA increased hepatic glycogen synthesis in ethanol-induced rats.

### MFA Inhibited Gluconeogenesis-Related Enzymes in Ethanol-Induced Rats

**Figure 6:**
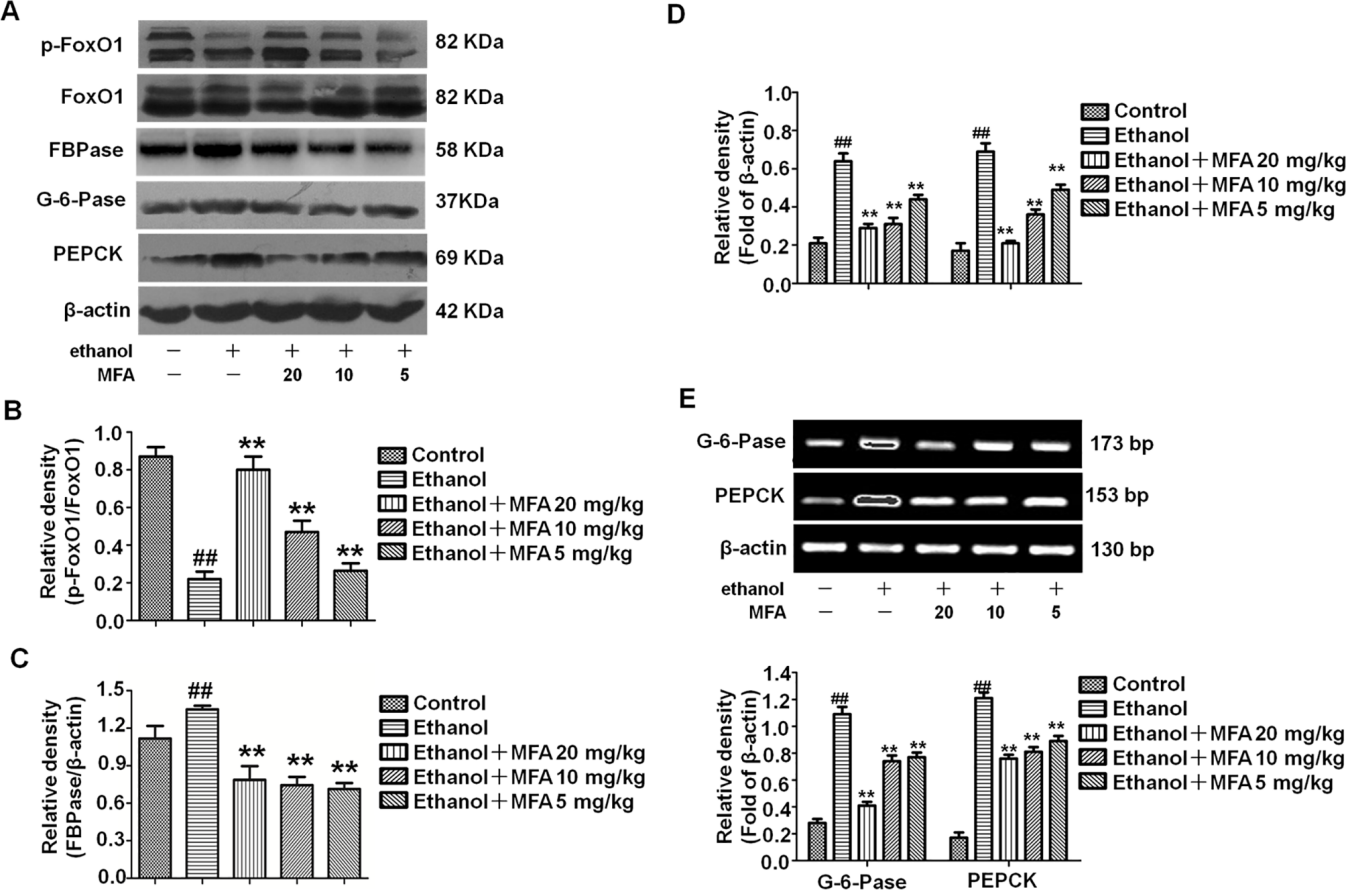
Effects of MFA on gluconeogenesis-related enzymes in livers of ethanol-induced rats. **(A)** Representative immunoblots of FoxO1, G-6-Pase, and PEPCK in rat liver tissues. **(B)** Levels of FoxO1 phosphorylation. **(C)** Levels of FBPase. **(D)** Levels of G-6-Pase and PEPCK. **(E)** mRNA expression of G-6-Pase and PEPCK. Data are mean ± SD of at least three separate experiments (n = 3). ^##^ *p* < 0.01, compared to the control group; **p < 0.01, compared to the ethan group.

Western blotting analysis of liver tissues demonstrated that ethanol significantly decreased the expression levels of p-FoxO1, which were notably increased in rats treated with MFA (*p* < 0.01; **Figures 6A, B**). As shown in **Figures 6C–E**, the expression levels of PEPCK, G-6-Pase, and FBPase were obviously increased in ethanol-induced rats compared to normal rats (*p* < 0.01); MFA treatment significantly down-regulated the increased expression levels of PEPCK, G-6-Pase, and FBPase (*p* < 0.01). Collectively, these results indicated that MFA inhibited the enzymatic capacity for gluconeogenesis in ethanol-induced rats.

### MFA Suppressed SREBP1 Over-Expression and TG Levels in Ethanol-Induced Rats

**Figure 7:**
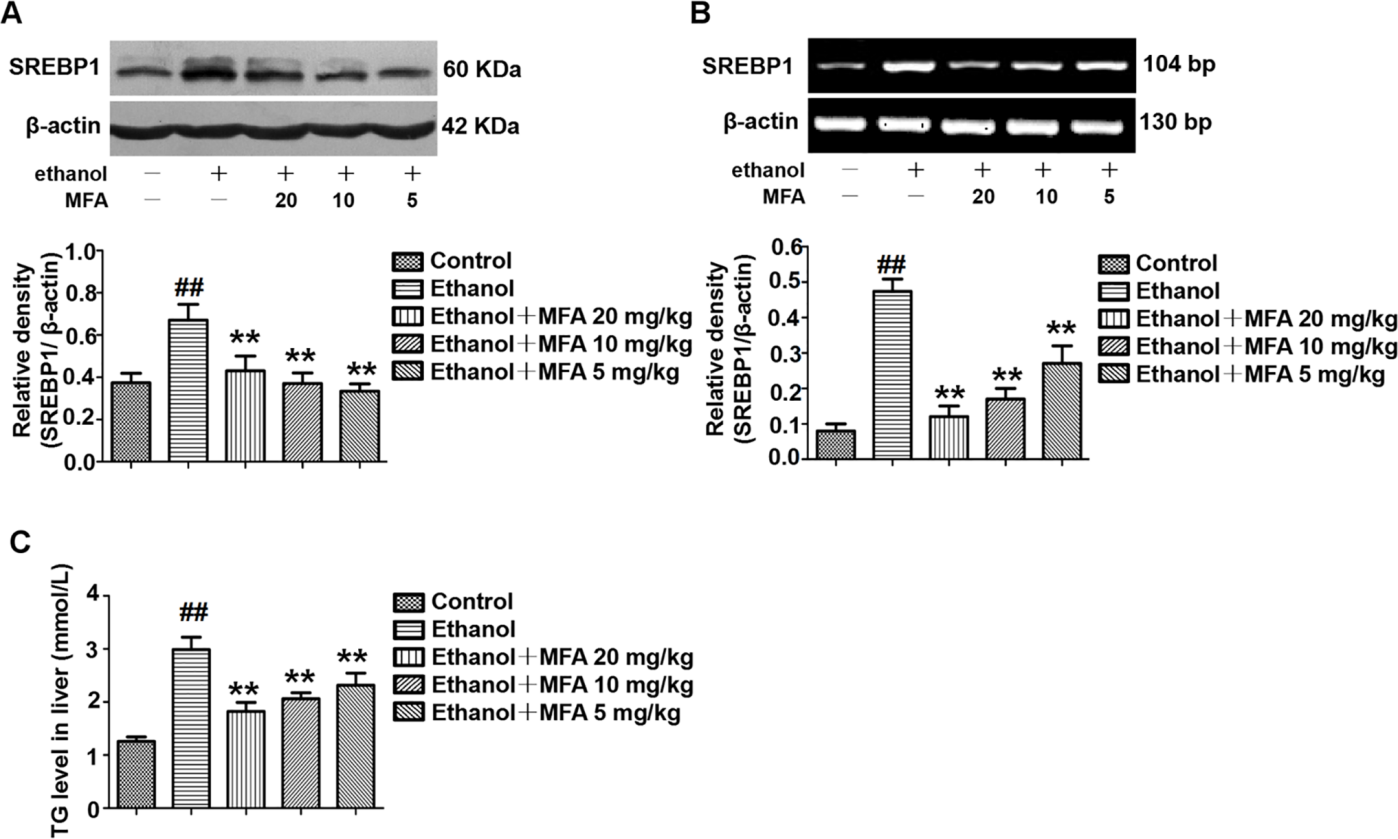
Effects of MFA on hepatic steatosis in ethanol-induced rats. **(A)** Representative immunoblots and levels of SREBP1 in rat liver tissues. **(B)** mRNA expression levels of SREBP1 in rat liver tissues. **(C)** TG levels in rat liver tissues. Data are mean ± SD of at least three separate experiments (n = 3). ^##^*p* < 0.01, compared to the control group; **p < 0.01, compared to the ethanol group.

As shown in **Figures 7A, B**, protein and mRNA expression levels of SREBP1 were significantly increased in rats induced by ethanol compared to the control rats (*p* < 0.01). However, the high expression levels of SREBP1 in ethanol-induced rats were notably down-regulated by MFA treatment (*p* < 0.01). In addition, TG levels were increased in rats in the ethanol group compared to the control group, which were rectified by MFA treatment (*p* < 0.01; **Figure 7C**). Taken together, these findings suggested that MFA decreased the expression of SREBP1 and TG levels in ethanol-induced rats.

### Effect of MFA on the Cell Viability of L-02 Cells

**Figure 8:**
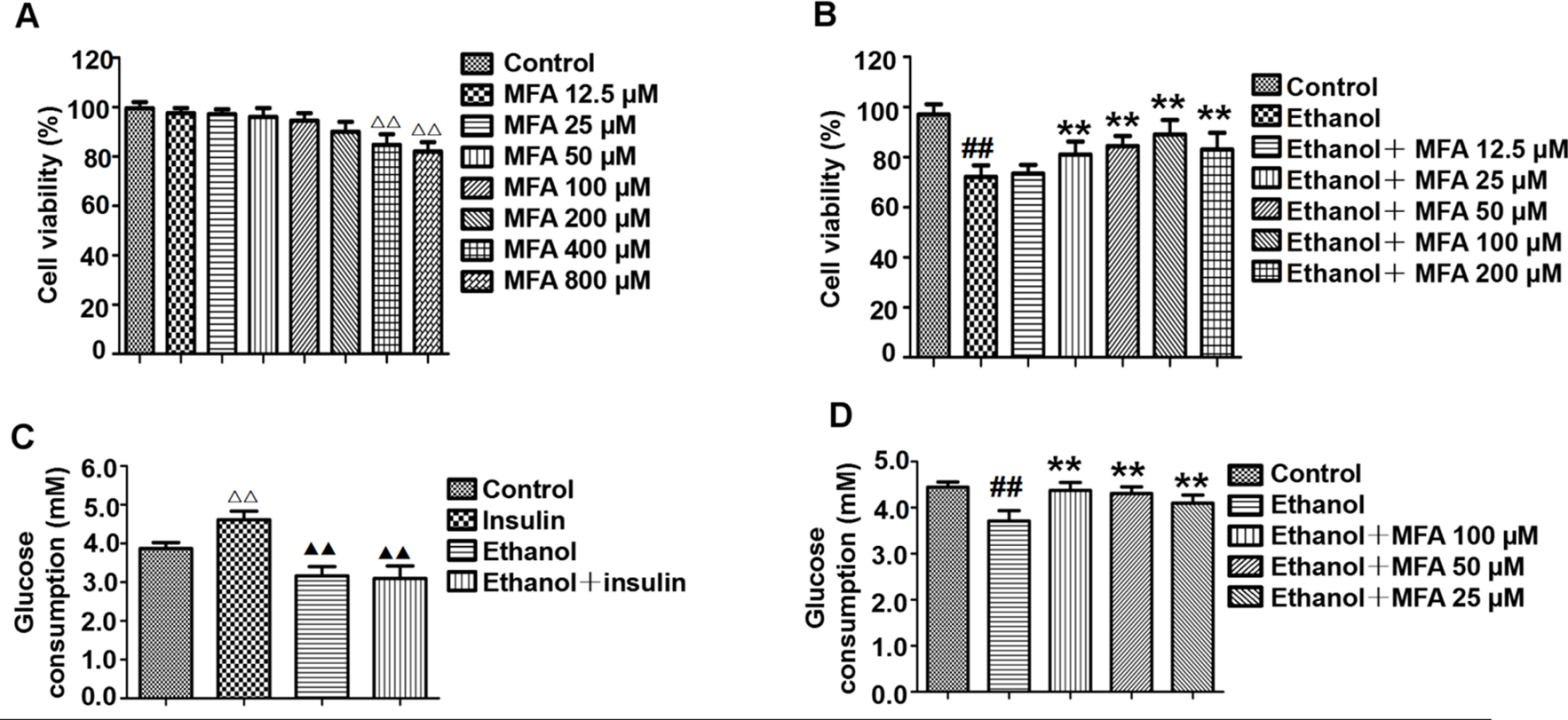
Effects of MFA on cell viability and glucose consumption in ethanol-induced L-02 cells. **(A)** Cell viability of various concentrations of MFA (12.5–800 μM) on L-02 cells. **(B)** Cell viability of 200 mM ethanol, 100 nM insulin, and 12.5 to 200 μM MFA on L-02 cells. **(C)** Glucose consumption in L-02 cells induced by 200 mM ethanol and/or 100 nM insulin. **(D)** Glucose consumption in L-02 cells. Data are mean ± SD of at least three separate experiments (n = 3). ##p < 0.01, compared to the control group; **p < 0.01, compared to the ethanol group. ^△△^p < 0.01, compared to the control group; ^△△^p < 0.01, compared to the insulin group.

Cell viability was assessed using the MTT assay. As shown in **Figure 8A**, compared to the control group, there was no significant difference in cell viability after treatment with 12.5 to 200 μM MFA for 24 h. However, the cell viability of L-02 cells treated with 400 and 800 μM MFA was reduced to less than 85% (*p* < 0.01). As shown in **Figure 8B**, the cell viability of L-02 cells was decreased after ethanol exposure, which was increased after MFA treatment. This difference was statistically significant at MFA concentrations from 25 to 200 μM (*p* < 0.01). However, 12.5 μM MFA did not notably improve the lower cell viability in ethanol-induced L-02 cells. Interestingly, the cell viability in the 200 μM MFA group was lower than in the 100 μM MFA group. Therefore, MFA concentrations of 25, 50, and 100 µM were chosen in subsequent experiments.

### MFA Enhanced Glucose Consumption in Ethanol-Induced L-02 Cells

As shown in **Figure 8C**, insulin treatment significantly increased glucose consumption in L-02 cells compared to the control group (*p* < 0.01). However, there was no obvious difference between the ethanol group and the ethanol + insulin group. In addition, compared to the insulin group, glucose consumption was notably decreased after treatment with ethanol and ethanol + insulin (*p* < 0.01). In addition, decreased glucose consumption in ethanol-induced L-02 cells was significantly up-regulated with MFA treatment in a dose-dependent manner (*p* < 0.01; **Figure 8D**). Taken together, these results suggested that MFA could enhance glucose consumption in ethanol-induced L-02 cells.

### MFA Ameliorated the Insulin Signaling Pathway in Ethanol-Induced L-02 Cells

**Figure 9:**
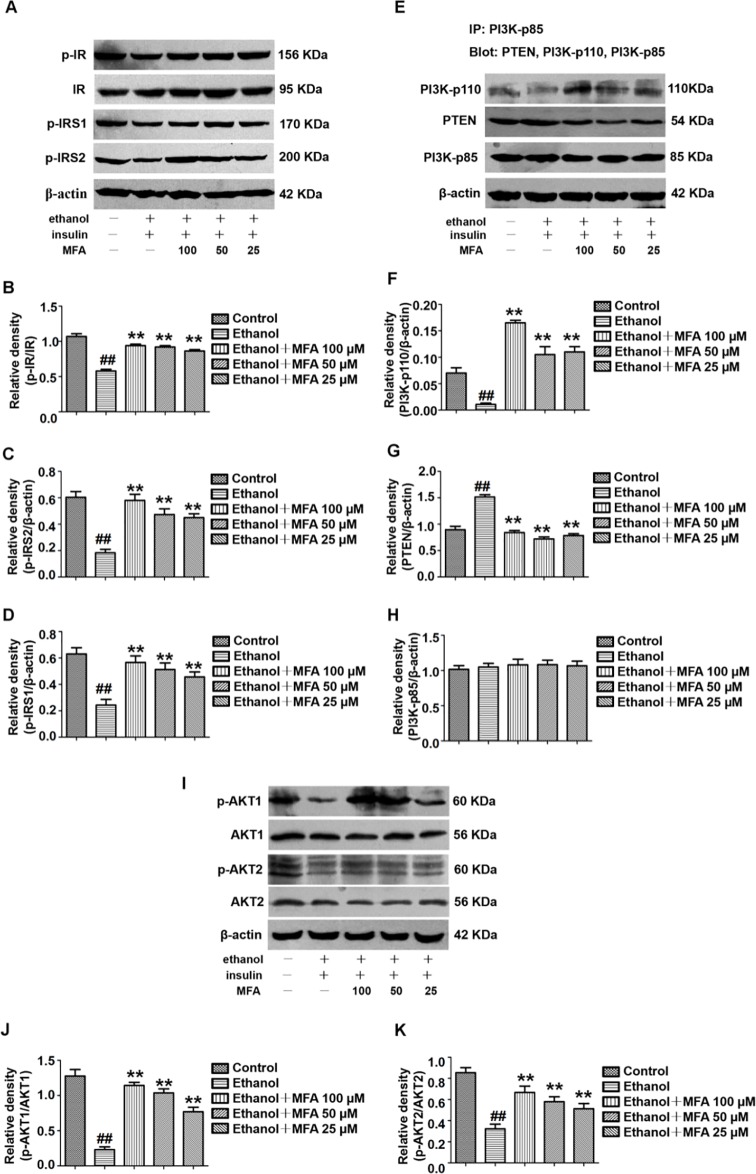
Effects of MFA on hepatic impairment of the insulin signaling pathway in ethanol-induced L-02 cells. **(A)** Representative immunoblots of IR, IRS1, and IRS2 in L-02 cells. **(B)** Levels of IR phosphorylation. **(C)** Levels of IRS1 phosphorylation. **(D)** Levels of IRS2 phosphorylation. **(E)** Representative immunoblots of PI3K and PTEN in L-02 cells. **(F)** Levels of PI3K-p110. **(G)** Levels of PTEN. **(H)** Levels of PI3K-p85. **(I)** Representative immunoblots of AKT1 and AKT2 in L-02 cells. **(J)** Level of AKT1 phosphorylation. **(K)** Levels of AKT2 phosphorylation. Data are mean ± SD of at least three separate experiments (n = 3). ^##^*p* < 0.01, compared to the control group; **p < 0.01, compared to the ethanol group.

In this study, the effect of MFA on the expression of major genes in the PI3K/Akt pathway in insulin-resistant L-02 cells was detected by Western blotting. The results indicated that ethanol exposure significantly decreased the protein expression levels of p-IR, p-IRS1, and p-IRS2 in ethanol-induced L-02 cells, which were dose-dependently increased after MFA treatment (*p* < 0.01; **Figures 9A–D**). Additionally, the protein expression levels of PI3K-p110 combined with PI3K-p85 were significantly lower in ethanol-induced rats than in normal L-02 cells (*p* < 0.01). In contrast, the protein expression levels of PTEN combined with PI3K-p85 were significantly increased in ethanol-induced L-02 cells than that in control L-02 cells (p < 0.01). MFA administration notably up-regulated the PI3K-p110 expression but down-regulated PTEN expression in ethanol-induced L-02 cells (*p* < 0.01; **Figures 9E–H**). Moreover, the results also suggested that MFA treatment markedly increased the expression levels of p-AKT1 and p-AKT2 in ethanol-induced L-02 cells (*p* < 0.01; **Figures 9I–K**). Collectively, these results indicated that MFA ameliorated the impairment of the hepatic insulin signaling pathway in ethanol-induced L-02 cells.

### MFA Improved the Reduction of Glycogen Synthesis and GLUT in Ethanol-Induced L-02 Cells

**Figure 10:**
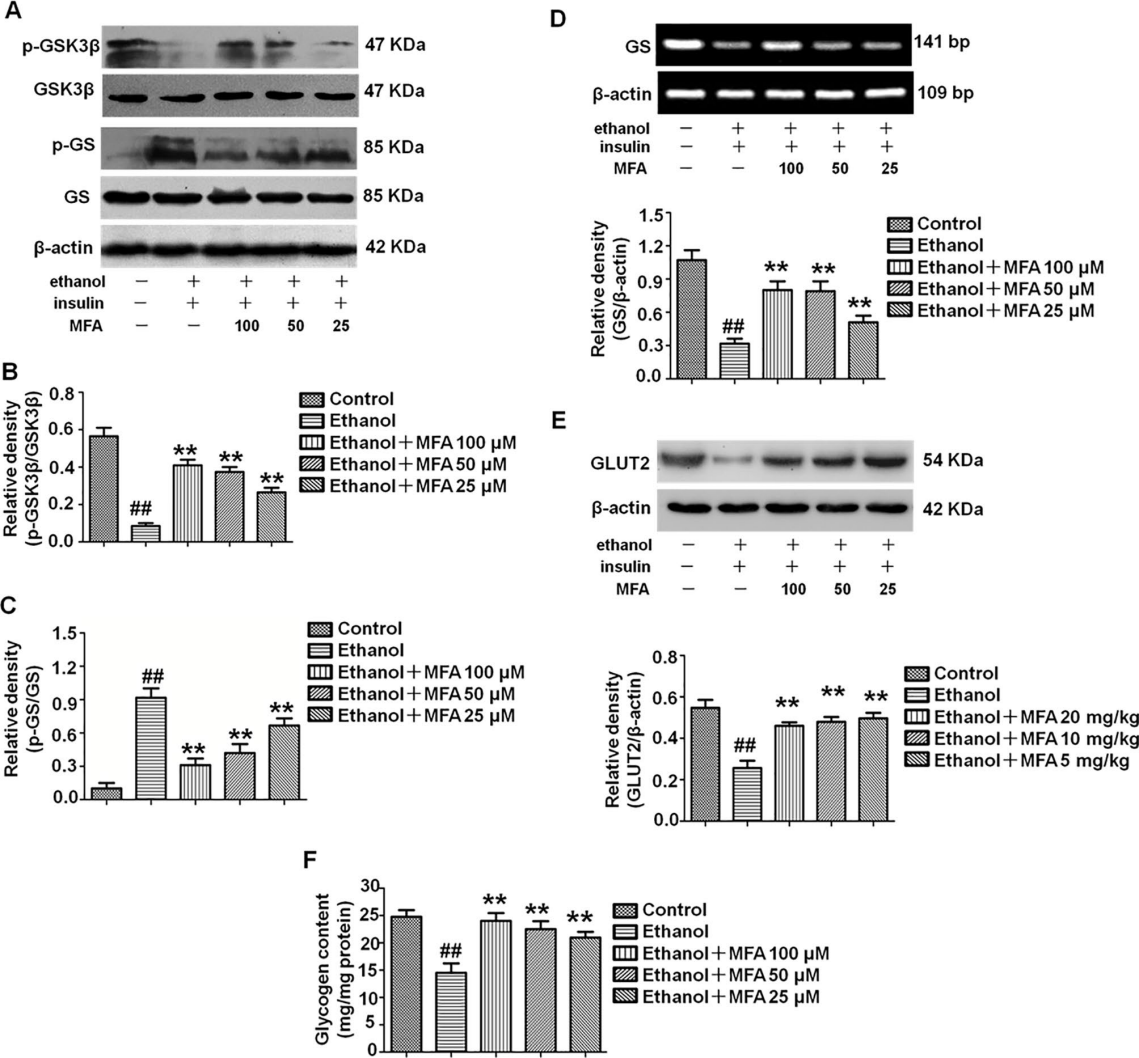
Effects of MFA on glycogen synthesis and GLUT2 in ethanol-induced L-02 cells. **(A)** Representative immunoblots of GSK3β and GS in L-02 cells. **(B)** Levels of GSK3β phosphorylation. **(C)** Levels of GS phosphorylation. **(D)** mRNA expression of GS. **(E)** Protein expression of GLUT2. **(F)** Glycogen content in L-02 cells. Data are mean ± SD of at least three separate experiments (n = 3). ^##^*p* < 0.01, compared to the control group; **p < 0.01, compared to the ethanol group.

In the present study, we evaluated the effects of MFA on glycogen synthesis by Western blotting and RT-PCR. As shown in **Figures 10A–C**, the protein expression levels of p-GSK3β were significantly decreased and those of p-GS were notably increased in ethanol-induced L-02 cells, which were both reversed with MFA treatment (*p* < 0.01). In addition, mRNA expression levels of GS were significantly lower in ethanol-induced L-02 cells than those in control L-02 cells (*p* < 0.01); MFA treatment significantly enhanced the mRNA expression levels of GS in L-02 cells induced by ethanol (*p* < 0.01; **Figure 10D**). Moreover, the decreased protein expression of GLUT2 in ethanol-induced L-02 cells was up-regulated by MFA treatment (*p* < 0.01; **Figure 10E**). Furthermore, ethanol induced a marked reduction of glycogen content in L-02 cells (*p* < 0.01). However, MFA treatment prevented the reduction of glycogen content in ethanol-induced L-02 cells (*p* < 0.01; **Figure 10F**). These results indicated that MFA enhanced glycogen synthesis in ethanol-induced L-02 cells.

### MFA Inhibited Hepatic Gluconeogenesis-Related Enzymes in Ethanol-Induced L-02 Cells

**Figure 11:**
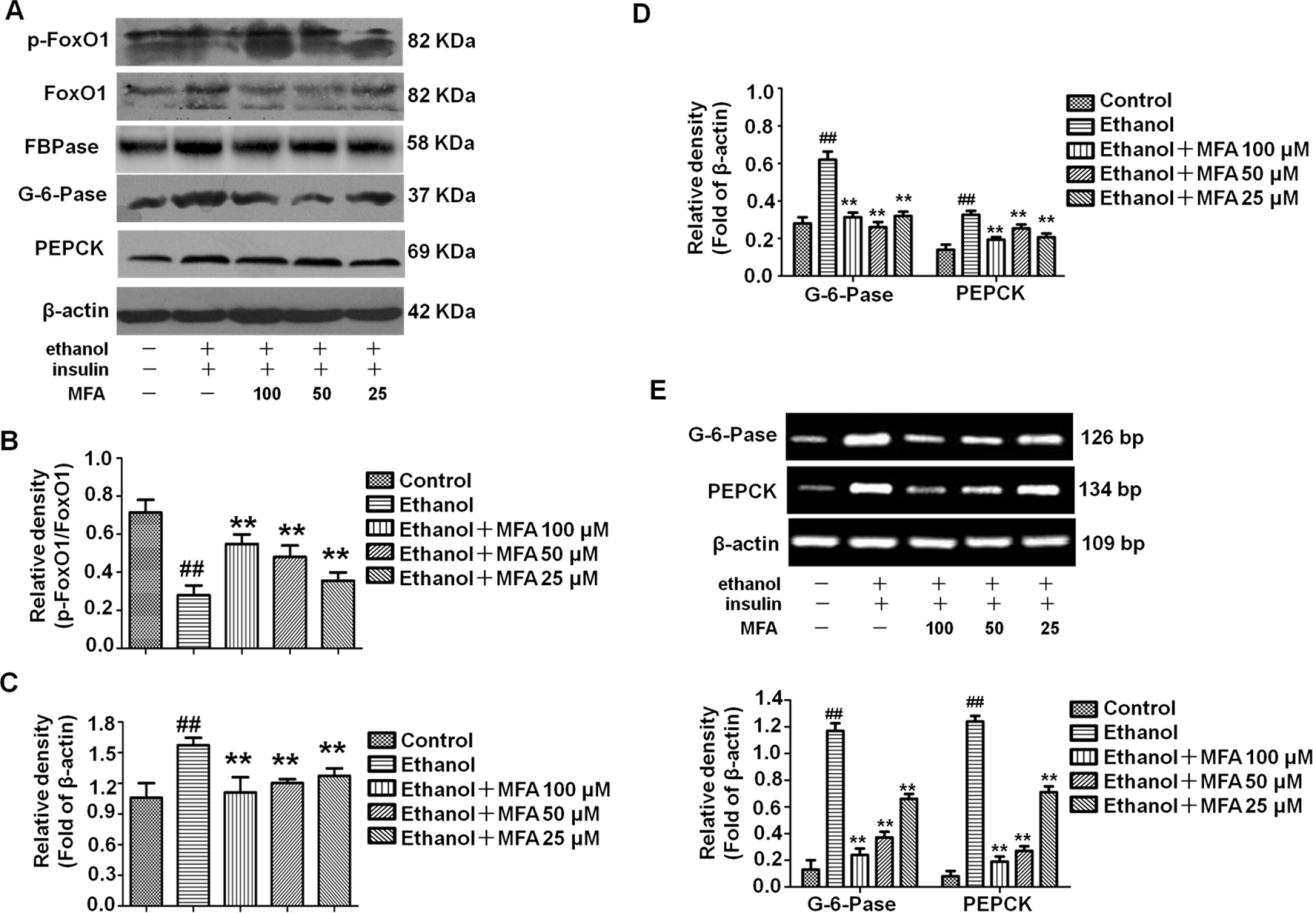
Effects of MFA on gluconeogenesis-related enzymes in ethanol-induced L-02 cells. **(A)** Representative immunoblots of FoxO1, G-6-Pase, and PEPCK in L-02 cells. **(B)** Levels of FoxO1 phosphorylation. **(C)** Levels of FBPase. **(D)** Levels of G-6-Pase and PEPCK. **(E)** mRNA expression of G-6-Pase and PEPCK. Data are mean ± SD of at least three separate experiments (n = 3). ^##^*p* < 0.01, compared to the control group; **p < 0.01, compared to the ethanol group.

To investigate the effect of MFA on the enzymatic capacity for gluconeogenesis in alcohol-induced L-02 cells, the expression levels of FoxO1, PEPCK, FBPase, and G-6-Pase were determined by Western blotting and RT-PCR. As shown in **Figures 11A, B** the protein expression levels of p-FoxO1 were significantly decreased in ethanol-induced L-02 cells compared to control L-02 cells, which could be increased by MFA treatment (*p* < 0.01). Furthermore, ethanol exposure clearly up-regulated the expression levels of FBPase, PEPCK, and G-6-Pase in L-02 cells, which were suppressed by MFA treatment (*p* < 0.01; **Figures 11C–E**). Taken together, these data showed that MFA might facilitate the phosphorylation of FoxO1 and suppress the expression of PEPCK, FBPase, and G-6-Pase in ethanol-induced L-02 cells.

### MFA Suppressed SREBP1 Over-Expression and TG Levels in Ethanol-Induced L-02 Cells

**Figure 12:**
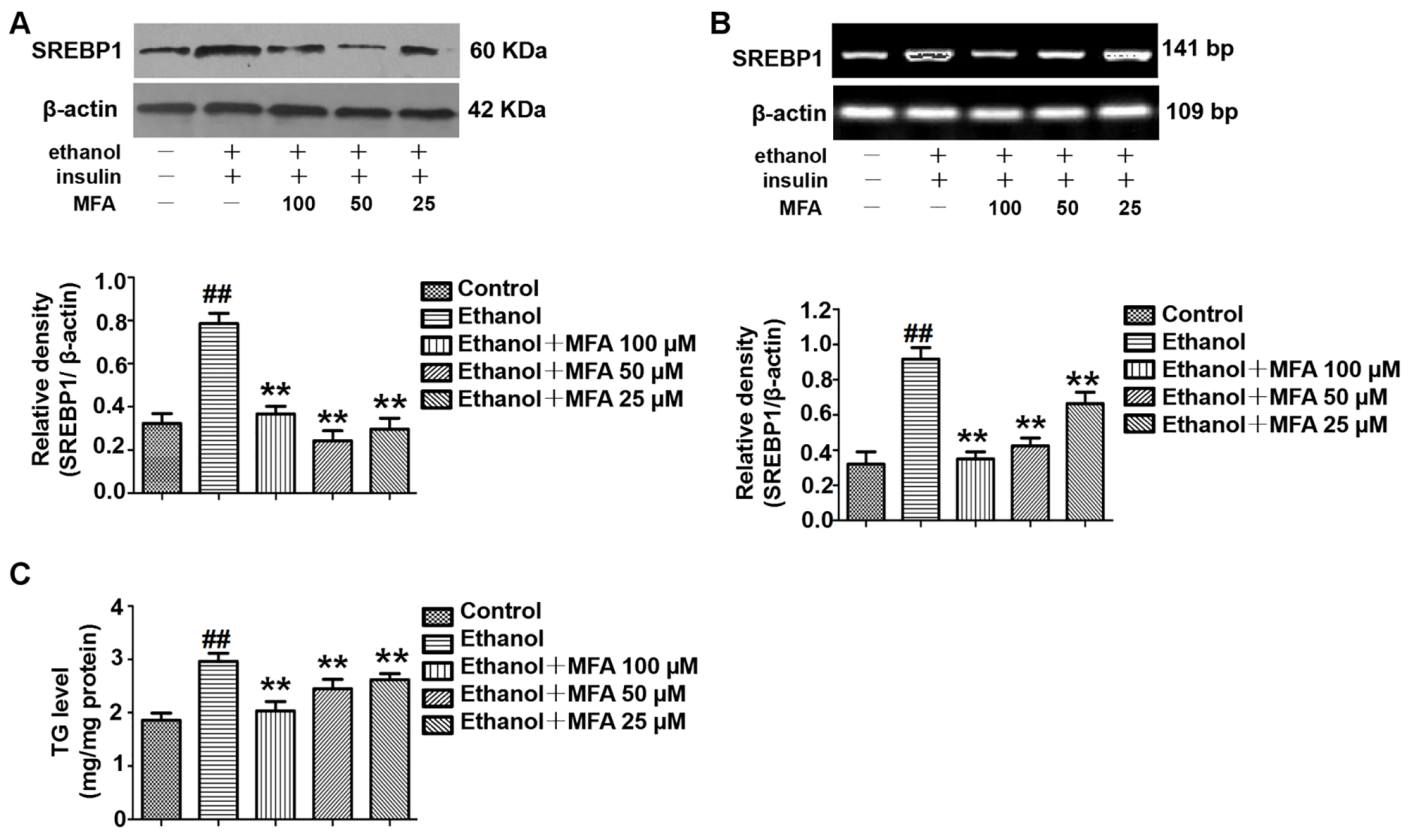
Effects of MFA on lipid deposition in ethanol-induced L-02 cells. **(A)** Representative immunoblots and levels of SREBP1 in L-02 cells. **(B)** mRNA expression levels of SREBP1 in L-02 cells. **(C)** TG levels in L-02 cells. Data are mean ± SD of at least three separate experiments (n = 3). ^##^*p* < 0.01, compared to the control group; **p < 0.01, compared to the ethanol group.

To investigate the expression of SREBP1 in ethanol-induced L-02 cells, we performed Western blotting and RT-PCR to detect the protein and mRNA expression levels of SREBP1. As shown in **Figures 12A, B**, compared to the control group, the expression levels of SREBP1 were significantly increased in L-02 cells in the ethanol group, and they were notably down-regulated in the MFA groups (*p* < 0.01). Consistent with these results, we evaluated intracellular TG levels and found that MFA significantly inhibited ethanol-induced increase in intracellular TG levels (*p* < 0.01; **Figure 12C**). These results suggested that MFA suppressed SREBP1 over-expression and decreased intracellular TG levels in ethanol-induced L-02 cells.

## Discussion

Recent epidemiological studies have found that alcohol consumption is closely related to insulin resistance, insulin secretion dysfunction, and a high incidence of metabolic syndrome (Åberg et al., 2018). In this study, we used an ethanol-induced ALD rat model and L-02 cell model to explore the beneficial metabolic effects and possible molecular mechanisms of MFA on ethanol-induced hepatic insulin resistance. The main findings of this study were as follows: 1) MFA reduced the enzymatic capacity for gluconeogenesis and increased glycogen synthesis in alcohol-induced rats, 2) MFA reduced the enzymatic capacity for gluconeogenesis and increased glycogen synthesis in ethanol-induced L-02 cells, and 3) our *in vivo* and *in vitro* studies showed for the first time that MFA attenuated ethanol-induced hepatic insulin resistance at least partially by enhancing the PI3K/AKT signaling pathway.

The animal model of ALD is the primary choice for most researchers who study chronic alcohol-induced liver disease. We developed an *in vivo* model of ALD by feeding SD rats liquid Lieber-DeCarli diet for 16 weeks to study chronic ethanol-induced hepatic insulin resistance. In this study, we observed that the weight of ALD rats was significantly decreased, the liver weight, spleen weight, and serum transaminase levels were significantly increased, and liver histopathological lesions were obvious. Consistent with previous reports (Sun et al., 2017; Xiao et al., 2017), these results indicated that the ALD model has been successfully established. Correspondingly, the significantly increased TG levels in rat livers in the model group reflected lipid metabolism disorder (Liu et al., 2014). Interestingly, our results were consistent with those of Xu et al., (2016). We observed no significant difference in fasting serum glucose levels in rats in each group, but the fasting insulin levels of rats in the model group were significantly higher than in the normal group. This finding indicated that ALD rats needed more insulin to control their blood glucose, and the hypoglycemic effect of insulin worsened, resulting in insulin resistance. Long-term high insulin levels will increase the body’s tolerance to insulin and reduce its sensitivity to insulin. Simultaneously, increased blood glucose and the development of diabetes could easily occur. However, research by Luo et al. showed that fasting serum glucose levels were increased in ethanol-induced insulin-resistant rats (Luo et al., 2017). These contradictory results might be attributed to the different methods and time of modeling, leading to different degrees of insulin resistance. As expected, after treatment with MFA in ALD rats, MFA not only alleviated the increase in the visceral index, increase in serum transaminase levels, and liver pathological damage in ALD rats, but it also significantly improved the lipid metabolism disorder and insulin resistance in ALD rats. In addition, *in vitro*, the MTT assay was used to confirm that the dosage of ethanol, insulin, and MFA used in this study was not toxic or deleterious to L-02 cells. Glucose consumption and intracellular glycogen content in ethanol-induced L-02 cells were also clearly decreased. The intracellular TG levels were also increased in ethanol-induced L-02 cells. In MFA-treated groups, glucose consumption and glycogen content in L-02 cells showed a tendency to increase in a dose-dependent manner, whereas TG levels were reduced, probably because MFA enhanced glucose metabolism and improved abnormal lipid metabolism and insulin resistance in ethanol-induced L-02 cells. Taken together, both *in vivo* and *in vitro* findings have shown that MFA has significant beneficial effects in alleviating alcohol-induced liver damage, lipid metabolism disorders, and insulin resistance.

Damage to the hepatic insulin signaling pathway can lead to insulin resistance (DeFronzo and Tripathy, 2009). There is increasing evidence that the PI3K/AKT pathway is one of the major pathways responsible for ethanol-induced insulin resistance (Sharma et al., 2015). A previous study has shown that ethanol inhibits the binding of insulin to the IR outside the hepatocyte membrane, leading to a decrease in IR phosphorylation, which in turn weakens IRS phosphorylation and inhibits the signaling cascade downstream of insulin (Carr and Correnti, 2015). The PI3K/AKT signaling pathway is regulated by the IRS and is one of the key pathways responsible for insulin regulation of the blood glucose balance (Li et al., 2016; Choi et al., 2018). Studies have shown that, using gene knockout and other techniques, functional defects in PI3K in regulatory subunit p85 and catalytic subunit p110 could all lead to disorders of glucose and lipid metabolism (Nelson et al., 2014; Winnay et al., 2014). AKT phosphorylation and activation can be controlled by PI3K and PTEN (Carr and Correnti, 2015). The p85 regulatory subunit of PI3K is the target of PTEN protein phosphatase activity (He et al., 2010). The combination of PI3K-p85 and PI3K-p110 induces the conversion of PIP2 into PIP3, which activates its target gene AKT through the phosphorylation of AKT to exert its biological effects; PTEN inhibits the phosphorylation of AKT by inducing the conversion of PIP3 to PIP2 by binding to PI3K-p85 (Anderson, 2010). Previous studies have shown that acute ethanol exposure both *in vitro* and *in vivo* can rapidly increase the binding of PTEN to PI3K-p85, leading to reduced AKT phosphorylation and inhibited biological activity (Yeon et al., 2003; He et al., 2007; Derdak et al., 2011). Similarly, in the current study, we found that the expression levels of p-IR, p-IRS1, and p-IRS2 in ethanol-induced L-02 cells and SD rat livers were significantly reduced. In addition, IP results showed that ethanol increased the production of PTEN and PI3K-p85 complexes in L-02 cells and SD rat livers, whereas PI3K-p110 and PI3K-p85 complexes were reduced. Furthermore, ethanol also inhibited the phosphorylation of AKT1 and AKT2 in L-02 cells and rat liver. However, these inhibitory effects of ethanol on the hepatic insulin signaling pathway were all reversed by MFA treatment. Therefore, the *in vivo* and *in vitro* results showed that MFA ameliorated the inhibition of ethanol on the PI3K/AKT signaling pathway.

Excessive hepatic glucose production is closely related to insulin resistance. It is well known that PEPCK and G-6-Pase are the rate-limiting enzymes in liver gluconeogenesis (Liu et al., 2015). A study has shown that insulin can increase the expression of FoxO1 through the phosphorylation and inactivation of FoxO1 and inhibit the expression of its downstream target genes PEPCK and G-6-Pase to reduce gluconeogenesis (Matsumoto et al., 2007). In addition, FBPase is the key enzyme for gluconeogenesis, as it irreversibly mediates the splitting of FBPase into fructose 6-phosphate and inorganic phosphate (Paksu et al., 2011). It was reported that insulin can reduce gluconeogenesis by inhibiting the expression and activity of FBPase by reducing the action of glucagon (El-Maghrabi et al., 1991). Our present results showed that the phosphorylation of FoxO1 was reduced and the expression levels of PEPCK, G-6-Pase, and FBPase increased in ethanol-induced L-02 cells and rat livers (Meng et al., 2013). GSK3β is a key enzyme involved in hepatic glucose metabolism, which activates GS by inhibiting GS phosphorylation and promotes glycogen synthesis (Shieh et al., 2009). GLUT-2 is the most important post-receptor signal transduction protein in insulin signal transduction pathway, which can transfer glucose from peripheral cells to hepatocytes for metabolism (Liu et al., 2019). We found that ethanol inhibited the phosphorylation of GSK3β and decreased the expression of GS and GLUT2 in L-02 cells and rat livers. SREBP1 is a transcription factor and is activated by AKT phosphorylation to induce hepatic lipogenesis (Ruiz et al., 2014). Our results suggested that ethanol could also up-regulate the expression levels of SREBP1 through the PI3K/AKT signaling pathway in L-02 cells and rat livers. However, after the administration of MFA, the expression levels of the gluconeogenic genes PEPCK, FBPase, G-6-Pase, and SREBP1 were down-regulated, the expression of GS and GLUT2 were up-regulated, and the phosphorylation levels of FoxO1 and GSK3β were increased in L-02 cells and rat livers. Therefore, both *in vitro* and *in vivo* experiments showed that MFA inhibited the ethanol-induced increase in PEPCK, G-6-Pase, FBPase, p-GS, and SREBP1 and increased the phosphorylation levels of FoxO1 and GSK3β. Therefore, both *in vivo* and *in vitro* results have shown that MFA improves ethanol-induced increase in the enzymatic capacity for gluconeogenesis, reduction in glycogen synthesis, and abnormal lipid metabolism.

In addition, we randomly selected three rat livers from the model group and mid-dose MFA group for transcriptome sequencing. According to the sequencing results, there were 252 DEGs in rat livers between the ethanol group and the MFA treatment group. In addition, glycolysis/gluconeogenesis/fatty acid metabolism pathways differed between the ethanol group and the MFA treatment group. These results partially validated our hypothesis, suggesting that MFA prevents alcohol-induced ALD by modulating glycolysis/gluconeogenesis/fatty acid metabolism. However, whether MFA improves ethanol-induced hepatic insulin resistance directly through the PI3K/AKT signaling pathway requires further investigation.

After long-term heavy drinking, ethanol can interfere with the expression or phosphorylation of IR, IRS1/2, PI3K, and Akt proteins. The activation of the PI3K-Akt pathway is inhibited, the sensitivity of hepatocytes to insulin is reduced, and hepatic insulin resistance is formed (Chen et al., 2018; Zeng et al., 2018). The body can only compensate by increasing insulin secretion. Excessive insulin secretion mediates the excessive activation of SREBP1, which increases the production of endogenous TG in the liver. The serious consequence is that TG accumulates in the liver. The large amount of fat that accumulated in liver cells further inhibits the sensitivity of hepatocytes to insulin and forms a vicious circle (Wang et al., 2013; Gu et al., 2015). MFA can improve alcohol-induced insulin resistance and abnormal glycolipid metabolism by activating the PI3K/AKT signaling pathway in the liver.

In conclusion, our *in vivo* and *in vitro* studies have shown that excessive ethanol exposure led to hepatic insulin resistance, which increased the enzymatic capacity for gluconeogenesis and lipogenesis and reduced glycogen synthesis by inhibiting the hepatic PI3K/AKT signaling pathway. Furthermore, we found for the first time that MFA had a certain degree of a therapeutic effect on ethanol-induced hepatic insulin resistance. In particular, MFA improved alcohol-induced insulin resistance at least in part through the PI3K/AKT signaling pathway. This study showed that MFA might be used as a potential drug to improve ethanol-induced insulin resistance. Future studies will be conducted to verify the exact anti-insulin resistance of MFA.

## Data Availability

The datasets analyzed in this manuscript are not publicly available. Requests to access the datasets should be directed to 31910753@qq.com.

## Ethics Statement

The animal study was reviewed and approved by the Experimental Animal Centre of Guilin Medical College, Guilin Medical University.

## Author Contributions

QC was responsible for writing articles. Y-WL was responsible for the idea. CY was responsible for *in vitro* experiments. YZ was responsible for doing *in vivo* experiments. LL was responsible for the revision and submission of articles.

## Funding

This research was supported by the National Natural Science Foundation of China (Nos. 81760669 and 81860660), the Guangxi Natural Science Foundation Project of Guangxi Province, China (Nos. 2017GXNSFAA198259 and 2018GXNSFDA281012), and the Innovation Project of Guangxi Graduate Education (No. YCSW2018211).

## Conflict of Interest Statement

The authors declare that the research was conducted in the absence of any commercial or financial relationships that could be construed as a potential conflict of interest.
